# Aqua­[1-(pyrazin-2-yl)ethanone oximato-κ^2^ *N*,*N*′][1-(pyrazin-2-yl)ethanone oxime-κ^2^ *N*,*N*′](thio­cyanato-κ*N*)nickel(II)

**DOI:** 10.1107/S1600536812024622

**Published:** 2012-06-13

**Authors:** Ting Pang, Jia-Cheng Liu, Zan Sun, Chao-Hu Xiao, Ping Cao

**Affiliations:** a Key Laboratory of Polymer Materials of Gansu Province, College of Chemistry and Chemical Engineering, Northwest Normal University, Lanzhou 730070, People’s Republic of China

## Abstract

In the title complex, [Ni(C_6_H_6_N_3_O)(NCS)(C_6_H_7_N_3_O)(H_2_O)] or [Ni(mpko)(SCN)(mpkoH)(H_2_O)] [where mpkoH = 1-(pyrazin-2-yl)ethanone oxime], the Ni^II^ cation is in a slightly distorted octa­hedral geometry, being coordinated in the equatorial plane by four N atoms from two different mpkoH ligands, one of which is deprotonated, and by one N atom from a thio­cyanate anion and one O atom from a water mol­ecule in the axial positions. There is an intra­molecular O—H⋯O hydrogen bond involving the oxime units of the two ligands. In the crystal, a three-dimensional supra­molecular architecture is formed by O—H⋯O and O—H⋯N hydrogen bonds.

## Related literature

For magnetic properties of related oxime complexes, see: Escuer *et al.* (2010 ▶); Radek *et al.* (1999 ▶, 2001 ▶); Spini (1973 ▶).
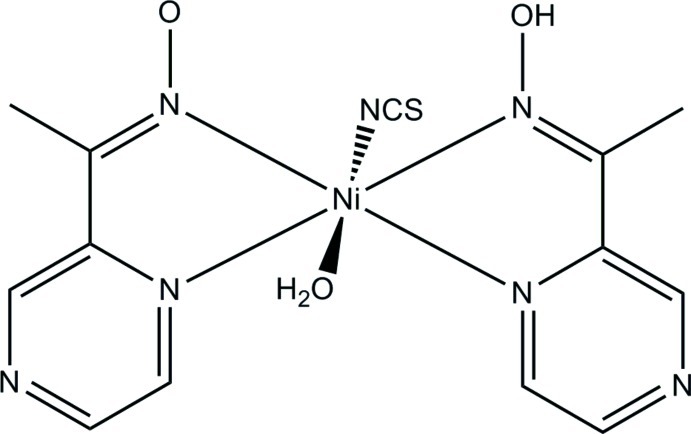


## Experimental

### 

#### Crystal data

[Ni(C_6_H_6_N_3_O)(NCS)(C_6_H_7_N_3_O)(H_2_O)]
*M* *_r_* = 408.09
Monoclinic, 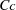

*a* = 11.917 (8) Å
*b* = 11.899 (8) Å
*c* = 12.354 (8) Å
β = 108.220 (5)°
*V* = 1664.1 (19) Å^3^
*Z* = 4
Mo *K*α radiation
μ = 1.32 mm^−1^
*T* = 296 K
0.36 × 0.32 × 0.29 mm

#### Data collection

Bruker APEXII CCD diffractometer
Absorption correction: multi-scan (*SADABS*; Bruker, 2000 ▶) *T*_min_ = 0.648, *T*_max_ = 0.701
5816 measured reflections
2809 independent reflections
2700 reflections with *I* > 2σ(*I*)
*R*_int_ = 0.017

#### Refinement

*R*[*F* ^2^ > 2σ(*F* ^2^)] = 0.020
*wR*(*F* ^2^) = 0.045
*S* = 1.05
2809 reflections
238 parameters
5 restraints
H atoms treated by a mixture of independent and constrained refinement
Δρ_max_ = 0.22 e Å^−3^
Δρ_min_ = −0.16 e Å^−3^
Absolute structure: Flack (1983 ▶), 1258 Friedel pairs
Flack parameter: 0.079 (9)

### 

Data collection: *APEX2* (Bruker, 2000 ▶); cell refinement: *SAINT* (Bruker, 2000 ▶); data reduction: *SAINT*; program(s) used to solve structure: *SHELXS97* (Sheldrick, 2008 ▶); program(s) used to refine structure: *SHELXL97* (Sheldrick, 2008 ▶); molecular graphics: *SHELXTL* (Sheldrick, 2008 ▶); software used to prepare material for publication: *SHELXTL*.

## Supplementary Material

Crystal structure: contains datablock(s) I, global. DOI: 10.1107/S1600536812024622/su2441sup1.cif

Structure factors: contains datablock(s) I. DOI: 10.1107/S1600536812024622/su2441Isup2.hkl

Additional supplementary materials:  crystallographic information; 3D view; checkCIF report

## Figures and Tables

**Table 1:** Hydrogen-bond geometry (Å, °)

*D*—H⋯*A*	*D*—H	H⋯*A*	*D*⋯*A*	*D*—H⋯*A*
O2—H1*O*⋯O3	0.89 (4)	1.63 (4)	2.505 (3)	167 (5)
O1—H1*W*⋯N2^i^	0.82	2.14	2.941 (4)	166
O1—H2*W*⋯O3^ii^	0.89 (2)	1.84 (2)	2.690 (3)	161 (2)

Symmetry codes: (i) 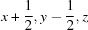; (ii) 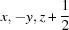.

**Fig. 1.**
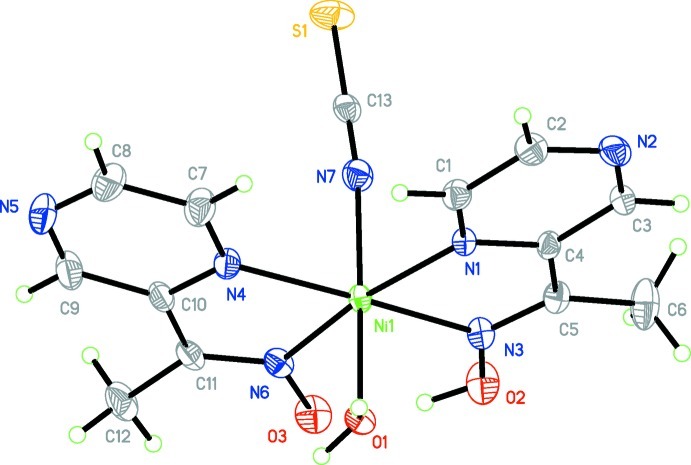
A view of the molecular structure of the title compound with the atom numbering. Displacement ellipsoids are drawn at the 30% probability level.

**Fig. 2.**
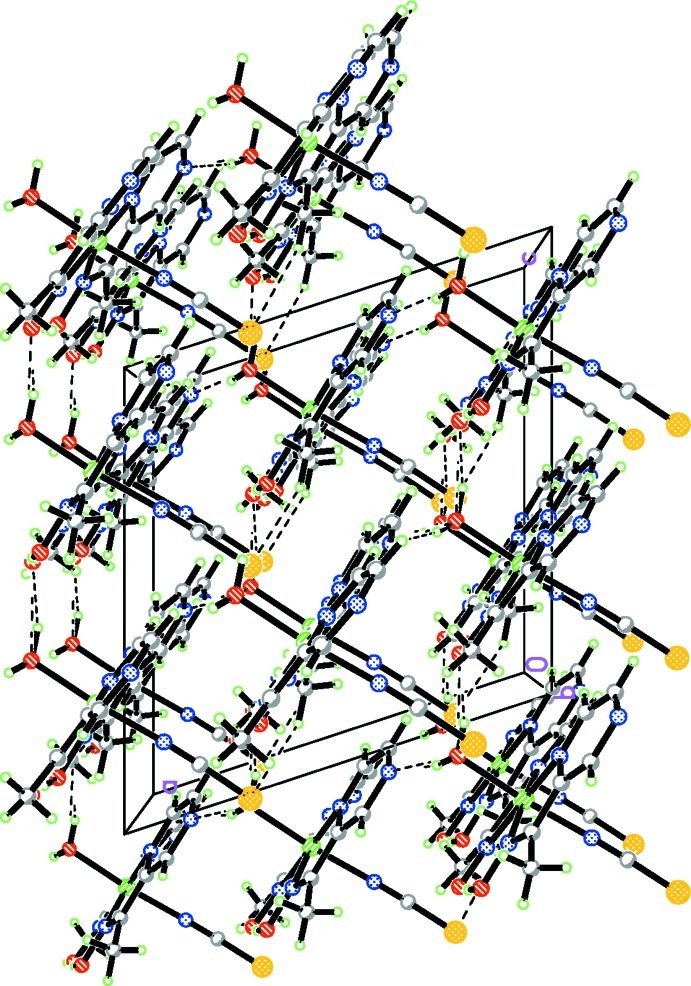
The crystal packing of the title compound, viewed along the b axis. The O-H···O and O-H···N hydrogen bonds are shown as dashed lines (see Table 1 for details).

## supplementary crystallographic information

### Comment

In the past decades, much attention has been paid to the design and synthesis of oximes complexes. Oximes can be feasibly synthesized by the Schiff base condensation of an aldehyde or ketone with hydroxylamine. To date, various oximate ligands as bridging ligands have been extensively explored for their great ability to form homo- and heterometallic polynuclear complexes, which can transmit magnetic exchange efficiently (Radek *et al.* 1999, 2001). Among oximate bridging ligands, *R*-substituted-pyridyloximes, (py)C(*R*)NOH, salycylaldoximes and *R*-saoH_2_ play an outstanding role to generate a great variety of polynuclear complexes which not only have aesthetically pleasing structures, but also possess interesting magnetic properties of single molecule magnet (SMM) and single chain magnet (SCM) behavior (Escuer *et al.*, 2010; Spini, 1973).

The title compound, Fig. 1, is a new nickel complex obtained by the reaction of nickel chloride hexahydrate with mpkoH (methyl pyrazine-2-yl ketoxime) in CH_3_OH solution. The Ni^II^ cation is in a slightly distorted octahedral geometry. The equatorial plane is defined by four N atoms from two mpkoH ligands - one of which is deprotonated, while the axial positions are occupied by one N atom from a SCN^-^ anion and one water O atom. There is an intramolecular O-H···O hydrogen bond (Table 1) involving the the oxime moieties of the two ligands.

In the crystal a three-dimensional supramolecular architecture is formed by O—H···O and O—H···N hydrogen bonds (Fig. 2 and Table 1).

### Experimental

The title complex was prepared by the addition of nickel chloride hexahydrate (23.9 mg, 0.1 mmol) to a CH_3_OH solution of methyl pyrazine-2-yl ketoxime (28 mg, 0.2 mmol); the pH was adjusted to 8 with 1*M* KSCN. Slow evaporation of the solvent gave red block-like crystals of the title compound, suitable for X-ray analysis, after several days at room temperature [Yield 31 mg, 77%]. Anal. Calc. for C_13_H_15_N_7_NiO_3_S: C, 38.26; H, 3.71; N, 24.03. Found: C, 36.93; H, 3.4; N, 25.1%.

### Refinement

The OH and water H atoms were located in a difference Fourier map. All except one of the water H atoms [H1W; constrained to be 0.82 Å with *U*_iso_(H) = 1.5U_eq_(O)], were freely refined. The C-bound H atoms were placed in calculated positions and refined as riding atoms: C—H = 0.93 and 0.96Å for CH and CH_3_ H atoms, respectively, with *U*_iso_(H) = k × U_eq_(C), where k = 1.5 for CH_3_ H atoms and = 1.2 for other H atoms.

### Crystal data

**Table:**

[Ni(C_6_H_6_N_3_O)(NCS)(C_6_H_7_N_3_O)(H_2_O)]	*F*(000) = 840
*M**_r_* = 408.09	*D*_x_ = 1.629 Mg m^−^^3^
Monoclinic, *C**c*	Mo *K*α radiation, λ = 0.71073 Å
Hall symbol: C -2yc	Cell parameters from 4663 reflections
*a* = 11.917 (8) Å	θ = 2.5–28.9°
*b* = 11.899 (8) Å	µ = 1.32 mm^−^^1^
*c* = 12.354 (8) Å	*T* = 296 K
β = 108.220 (5)°	Block, red
*V* = 1664.1 (19) Å^3^	0.36 × 0.32 × 0.29 mm
*Z* = 4	

### Data collection

**Table:**

Bruker APEXII CCD diffractometer	2809 independent reflections
Radiation source: fine-focus sealed tube	2700 reflections with *I* > 2σ(*I*)
Graphite monochromator	*R*_int_ = 0.017
φ and ω scans	θ_max_ = 25.5°, θ_min_ = 2.5°
Absorption correction: multi-scan (*SADABS*; Bruker, 2000)	*h* = −13→14
*T*_min_ = 0.648, *T*_max_ = 0.701	*k* = −14→14
5816 measured reflections	*l* = −14→14

### Refinement

**Table:**

Refinement on *F*^2^	Hydrogen site location: inferred from neighbouring sites
Least-squares matrix: full	H atoms treated by a mixture of independent and constrained refinement
*R*[*F*^2^ > 2σ(*F*^2^)] = 0.020	*w* = 1/[σ^2^(*F*_o_^2^) + (0.0195*P*)^2^] where *P* = (*F*_o_^2^ + 2*F*_c_^2^)/3
*wR*(*F*^2^) = 0.045	(Δ/σ)_max_ = 0.001
*S* = 1.05	Δρ_max_ = 0.22 e Å^−^^3^
2809 reflections	Δρ_min_ = −0.16 e Å^−^^3^
238 parameters	Extinction correction: *SHELXL97* (Sheldrick, 2008), Fc^*^=kFc[1+0.001xFc^2^λ^3^/sin(2θ)]^-1/4^
5 restraints	Extinction coefficient: 0.0035 (2)
Primary atom site location: structure-invariant direct methods	Absolute structure: Flack (1983), 1258 Friedel pairs
Secondary atom site location: difference Fourier map	Flack parameter: 0.079 (9)

### Special details

**Table:**

Geometry. Bond distances, angles etc. have been calculated using the rounded fractional coordinates. All su's are estimated from the variances of the (full) variance-covariance matrix. The cell esds are taken into account in the estimation of distances, angles and torsion angles
Refinement. Refinement of *F*^2^ against ALL reflections. The weighted *R*-factor *wR* and goodness of fit *S* are based on *F*^2^, conventional *R*-factors *R* are based on *F*, with *F* set to zero for negative *F*^2^. The threshold expression of *F*^2^ > σ(*F*^2^) is used only for calculating *R*-factors(gt) *etc*. and is not relevant to the choice of reflections for refinement. *R*-factors based on *F*^2^ are statistically about twice as large as those based on *F*, and *R*- factors based on ALL data will be even larger.

### Fractional atomic coordinates and isotropic or equivalent isotropic displacement parameters (Å^2^)

**Table:**

	*x*	*y*	*z*	*U*_iso_*/*U*_eq_	
Ni1	0.06769 (2)	0.04643 (2)	0.79647 (2)	0.0232 (1)	
S1	−0.29404 (7)	0.01219 (7)	0.48705 (6)	0.0505 (3)	
O1	0.22258 (19)	0.04631 (13)	0.93718 (17)	0.0297 (6)	
O2	0.1754 (2)	0.19519 (17)	0.65847 (17)	0.0455 (7)	
O3	0.21552 (17)	−0.01069 (15)	0.64998 (15)	0.0416 (6)	
N1	−0.00488 (18)	0.17283 (15)	0.87702 (17)	0.0243 (6)	
N2	−0.12050 (19)	0.35594 (17)	0.93569 (18)	0.0389 (7)	
N3	0.11228 (18)	0.19306 (17)	0.73314 (18)	0.0291 (7)	
N4	0.02311 (18)	−0.11543 (16)	0.83966 (17)	0.0308 (7)	
N5	−0.0404 (2)	−0.33939 (18)	0.8492 (3)	0.0609 (10)	
N6	0.14984 (18)	−0.05268 (16)	0.71043 (16)	0.0294 (6)	
N7	−0.0897 (2)	0.04323 (17)	0.6682 (2)	0.0364 (9)	
C1	−0.0646 (2)	0.1639 (2)	0.9510 (2)	0.0324 (8)	
C2	−0.1229 (2)	0.2544 (2)	0.9789 (2)	0.0376 (8)	
C3	−0.0582 (2)	0.36562 (18)	0.8631 (2)	0.0338 (8)	
C4	−0.0003 (2)	0.27569 (18)	0.83197 (19)	0.0262 (7)	
C5	0.0660 (2)	0.28514 (19)	0.7509 (2)	0.0309 (7)	
C6	0.0749 (3)	0.3925 (2)	0.6917 (2)	0.0552 (12)	
C7	−0.0488 (3)	−0.1484 (2)	0.8961 (2)	0.0452 (10)	
C8	−0.0807 (3)	−0.2595 (3)	0.8998 (3)	0.0552 (11)	
C9	0.0317 (3)	−0.3073 (2)	0.7932 (3)	0.0508 (10)	
C10	0.0655 (3)	−0.19532 (16)	0.7858 (3)	0.0332 (7)	
C11	0.1399 (2)	−0.1601 (2)	0.7182 (2)	0.0321 (8)	
C12	0.1990 (3)	−0.2437 (2)	0.6627 (3)	0.0485 (10)	
C13	−0.1750 (2)	0.03029 (18)	0.5946 (2)	0.0290 (8)	
H1	−0.06730	0.09470	0.98520	0.0390*	
H1O	0.200 (4)	0.125 (3)	0.654 (3)	0.092 (14)*	
H1W	0.26870	−0.00070	0.92730	0.0450*	
H2	−0.16540	0.24360	1.02960	0.0450*	
H2W	0.207 (2)	0.0450 (19)	1.0028 (13)	0.053 (9)*	
H3	−0.05340	0.43590	0.83190	0.0410*	
H6A	0.13750	0.43750	0.74000	0.0830*	
H6B	0.00160	0.43270	0.67490	0.0830*	
H6C	0.09130	0.37630	0.62200	0.0830*	
H7	−0.07890	−0.09500	0.93470	0.0540*	
H8	−0.13260	−0.27820	0.93960	0.0660*	
H9	0.06190	−0.36210	0.75640	0.0610*	
H12A	0.14020	−0.28170	0.60260	0.0730*	
H12B	0.24150	−0.29750	0.71840	0.0730*	
H12C	0.25290	−0.20530	0.63180	0.0730*	

### Atomic displacement parameters (Å^2^)

**Table:**

	*U*^11^	*U*^22^	*U*^33^	*U*^12^	*U*^13^	*U*^23^
Ni1	0.0290 (2)	0.0193 (1)	0.0225 (1)	0.0021 (2)	0.0098 (1)	−0.0009 (2)
S1	0.0319 (4)	0.0722 (5)	0.0404 (4)	0.0037 (3)	0.0012 (3)	−0.0099 (3)
O1	0.0305 (11)	0.0341 (11)	0.0258 (11)	0.0046 (7)	0.0106 (9)	−0.0009 (7)
O2	0.0666 (14)	0.0386 (11)	0.0455 (12)	0.0003 (10)	0.0382 (11)	0.0025 (9)
O3	0.0520 (11)	0.0481 (10)	0.0342 (10)	0.0031 (9)	0.0270 (9)	−0.0049 (8)
N1	0.0280 (11)	0.0209 (10)	0.0245 (10)	0.0003 (8)	0.0089 (9)	0.0004 (8)
N2	0.0336 (12)	0.0374 (11)	0.0453 (13)	0.0056 (9)	0.0119 (10)	−0.0114 (10)
N3	0.0369 (13)	0.0269 (11)	0.0259 (10)	0.0019 (9)	0.0135 (9)	0.0008 (8)
N4	0.0342 (12)	0.0252 (11)	0.0340 (11)	−0.0011 (9)	0.0122 (9)	−0.0011 (9)
N5	0.0601 (17)	0.0271 (12)	0.088 (2)	−0.0061 (12)	0.0124 (16)	0.0101 (13)
N6	0.0315 (11)	0.0319 (10)	0.0236 (10)	0.0050 (9)	0.0071 (9)	−0.0050 (8)
N7	0.0376 (18)	0.0334 (14)	0.0363 (16)	0.0025 (9)	0.0087 (13)	−0.0069 (9)
C1	0.0346 (14)	0.0312 (13)	0.0341 (14)	−0.0014 (11)	0.0145 (12)	0.0001 (11)
C2	0.0380 (15)	0.0403 (14)	0.0385 (14)	0.0008 (11)	0.0178 (12)	−0.0066 (11)
C3	0.0327 (14)	0.0237 (12)	0.0423 (14)	0.0036 (10)	0.0080 (11)	0.0003 (10)
C4	0.0294 (13)	0.0204 (11)	0.0245 (12)	−0.0015 (10)	0.0024 (10)	−0.0019 (9)
C5	0.0380 (14)	0.0262 (12)	0.0284 (12)	−0.0024 (11)	0.0104 (11)	0.0008 (9)
C6	0.096 (3)	0.0296 (13)	0.0501 (18)	−0.0041 (14)	0.0375 (18)	0.0044 (12)
C7	0.0537 (19)	0.0355 (15)	0.0549 (18)	−0.0051 (13)	0.0291 (16)	0.0012 (12)
C8	0.053 (2)	0.0441 (18)	0.072 (2)	−0.0113 (16)	0.0244 (18)	0.0159 (16)
C9	0.055 (2)	0.0264 (12)	0.0639 (18)	0.0060 (11)	0.0084 (18)	−0.0039 (14)
C10	0.0328 (13)	0.0239 (10)	0.0367 (14)	0.0079 (14)	0.0019 (10)	−0.0015 (13)
C11	0.0321 (14)	0.0296 (13)	0.0293 (13)	0.0109 (11)	0.0021 (11)	−0.0053 (10)
C12	0.055 (2)	0.0380 (16)	0.0482 (17)	0.0155 (14)	0.0098 (15)	−0.0156 (13)
C13	0.0308 (15)	0.0275 (12)	0.0317 (13)	0.0049 (10)	0.0141 (12)	−0.0034 (9)

### Geometric parameters (Å, º)

**Table:**

Ni1—O1	2.103 (3)	N7—C13	1.143 (3)
Ni1—N1	2.130 (2)	C1—C2	1.382 (4)
Ni1—N3	2.049 (2)	C3—C4	1.391 (3)
Ni1—N4	2.110 (2)	C4—C5	1.461 (4)
Ni1—N6	2.032 (2)	C5—C6	1.492 (4)
Ni1—N7	2.043 (3)	C7—C8	1.380 (4)
S1—C13	1.627 (3)	C9—C10	1.403 (3)
O2—N3	1.361 (3)	C10—C11	1.457 (4)
O3—N6	1.336 (3)	C11—C12	1.503 (4)
O1—H1W	0.8200	C1—H1	0.9300
O1—H2W	0.887 (18)	C2—H2	0.9300
O2—H1O	0.89 (4)	C3—H3	0.9300
N1—C1	1.327 (3)	C6—H6A	0.9600
N1—C4	1.353 (3)	C6—H6B	0.9600
N2—C2	1.325 (3)	C6—H6C	0.9600
N2—C3	1.336 (3)	C7—H7	0.9300
N3—C5	1.276 (3)	C8—H8	0.9300
N4—C10	1.346 (4)	C9—H9	0.9300
N4—C7	1.322 (4)	C12—H12A	0.9600
N5—C9	1.317 (5)	C12—H12B	0.9600
N5—C8	1.309 (5)	C12—H12C	0.9600
N6—C11	1.290 (3)		
			
O1—Ni1—N1	89.65 (8)	C3—C4—C5	123.5 (2)
O1—Ni1—N3	92.91 (8)	C4—C5—C6	122.6 (2)
O1—Ni1—N4	90.90 (7)	N3—C5—C4	114.0 (2)
O1—Ni1—N6	89.43 (8)	N3—C5—C6	123.4 (2)
O1—Ni1—N7	175.60 (9)	N4—C7—C8	122.2 (3)
N1—Ni1—N3	76.64 (8)	N5—C8—C7	122.3 (3)
N1—Ni1—N4	110.80 (8)	N5—C9—C10	123.9 (3)
N1—Ni1—N6	170.52 (8)	N4—C10—C9	118.6 (3)
N1—Ni1—N7	88.12 (9)	N4—C10—C11	118.0 (2)
N3—Ni1—N4	171.68 (8)	C9—C10—C11	123.3 (3)
N3—Ni1—N6	93.99 (8)	N6—C11—C12	123.7 (2)
N3—Ni1—N7	90.26 (9)	C10—C11—C12	121.8 (2)
N4—Ni1—N6	78.65 (8)	N6—C11—C10	114.5 (2)
N4—Ni1—N7	86.36 (8)	S1—C13—N7	178.2 (2)
N6—Ni1—N7	93.39 (9)	N1—C1—H1	119.00
Ni1—O1—H1W	109.00	C2—C1—H1	119.00
Ni1—O1—H2W	112.0 (15)	N2—C2—H2	119.00
H1W—O1—H2W	118.00	C1—C2—H2	119.00
N3—O2—H1O	107 (3)	N2—C3—H3	118.00
Ni1—N1—C4	111.85 (16)	C4—C3—H3	118.00
C1—N1—C4	117.1 (2)	C5—C6—H6A	109.00
Ni1—N1—C1	130.46 (15)	C5—C6—H6B	109.00
C2—N2—C3	115.8 (2)	C5—C6—H6C	109.00
O2—N3—C5	117.4 (2)	H6A—C6—H6B	110.00
Ni1—N3—C5	119.20 (18)	H6A—C6—H6C	109.00
Ni1—N3—O2	122.62 (15)	H6B—C6—H6C	109.00
Ni1—N4—C10	110.89 (18)	N4—C7—H7	119.00
C7—N4—C10	117.2 (2)	C8—C7—H7	119.00
Ni1—N4—C7	131.30 (17)	N5—C8—H8	119.00
C8—N5—C9	115.9 (3)	C7—C8—H8	119.00
Ni1—N6—O3	122.51 (14)	N5—C9—H9	118.00
Ni1—N6—C11	117.75 (17)	C10—C9—H9	118.00
O3—N6—C11	119.7 (2)	C11—C12—H12A	109.00
Ni1—N7—C13	173.0 (2)	C11—C12—H12B	109.00
N1—C1—C2	122.0 (2)	C11—C12—H12C	110.00
N2—C2—C1	122.3 (2)	H12A—C12—H12B	110.00
N2—C3—C4	123.2 (2)	H12A—C12—H12C	110.00
N1—C4—C3	119.7 (2)	H12B—C12—H12C	110.00
N1—C4—C5	116.9 (2)		
			
O1—Ni1—N1—C1	−86.7 (2)	C1—N1—C4—C5	179.8 (2)
N3—Ni1—N1—C1	−179.7 (2)	Ni1—N1—C4—C3	171.17 (18)
N4—Ni1—N1—C1	4.2 (2)	C1—N1—C4—C3	−0.8 (3)
N7—Ni1—N1—C1	89.6 (2)	C2—N2—C3—C4	1.0 (4)
O1—Ni1—N1—C4	102.77 (17)	C3—N2—C2—C1	0.1 (4)
N3—Ni1—N1—C4	9.69 (16)	Ni1—N3—C5—C6	−168.53 (19)
N4—Ni1—N1—C4	−166.41 (16)	O2—N3—C5—C6	1.9 (4)
N7—Ni1—N1—C4	−81.03 (17)	Ni1—N3—C5—C4	9.5 (3)
O1—Ni1—N3—O2	90.31 (19)	O2—N3—C5—C4	179.9 (2)
N1—Ni1—N3—O2	179.3 (2)	C10—N4—C7—C8	−0.5 (4)
N6—Ni1—N3—O2	0.7 (2)	Ni1—N4—C10—C11	5.1 (3)
N7—Ni1—N3—O2	−92.7 (2)	C7—N4—C10—C11	176.8 (3)
O1—Ni1—N3—C5	−99.8 (2)	C7—N4—C10—C9	−0.3 (4)
N1—Ni1—N3—C5	−10.86 (19)	Ni1—N4—C7—C8	169.3 (2)
N6—Ni1—N3—C5	170.6 (2)	Ni1—N4—C10—C9	−172.1 (3)
N7—Ni1—N3—C5	77.2 (2)	C8—N5—C9—C10	−0.1 (5)
O1—Ni1—N4—C7	97.6 (2)	C9—N5—C8—C7	−0.7 (5)
N1—Ni1—N4—C7	7.7 (3)	Ni1—N6—C11—C12	−177.9 (2)
N6—Ni1—N4—C7	−173.1 (2)	O3—N6—C11—C10	−179.7 (2)
N7—Ni1—N4—C7	−78.9 (2)	O3—N6—C11—C12	0.2 (4)
O1—Ni1—N4—C10	−92.1 (2)	Ni1—N6—C11—C10	2.3 (3)
N1—Ni1—N4—C10	177.9 (2)	N1—C1—C2—N2	−1.6 (4)
N6—Ni1—N4—C10	−2.9 (2)	N2—C3—C4—N1	−0.7 (4)
N7—Ni1—N4—C10	91.3 (2)	N2—C3—C4—C5	178.7 (2)
O1—Ni1—N6—O3	−86.70 (18)	N1—C4—C5—N3	−0.2 (3)
N3—Ni1—N6—O3	6.18 (18)	N1—C4—C5—C6	177.9 (2)
N4—Ni1—N6—O3	−177.73 (19)	C3—C4—C5—C6	−1.6 (4)
N7—Ni1—N6—O3	96.68 (18)	C3—C4—C5—N3	−179.7 (2)
O1—Ni1—N6—C11	91.28 (18)	N4—C7—C8—N5	1.1 (5)
N3—Ni1—N6—C11	−175.84 (19)	N5—C9—C10—C11	−176.4 (3)
N4—Ni1—N6—C11	0.25 (18)	N5—C9—C10—N4	0.6 (5)
N7—Ni1—N6—C11	−85.35 (19)	N4—C10—C11—C12	175.1 (3)
Ni1—N1—C1—C2	−168.28 (18)	C9—C10—C11—N6	171.9 (3)
C4—N1—C1—C2	1.9 (4)	C9—C10—C11—C12	−7.9 (5)
Ni1—N1—C4—C5	−8.3 (3)	N4—C10—C11—N6	−5.1 (4)

### Hydrogen-bond geometry (Å, º)

**Table:**

*D*—H···*A*	*D*—H	H···*A*	*D*···*A*	*D*—H···*A*
O2—H1*O*···O3	0.89 (4)	1.63 (4)	2.505 (3)	167 (5)
O1—H1*W*···N2^i^	0.82	2.14	2.941 (4)	166
O1—H2*W*···O3^ii^	0.89 (2)	1.84 (2)	2.690 (3)	161 (2)

Symmetry codes: (i) *x*+1/2, *y*−1/2, *z*; (ii) *x*, −*y*, *z*+1/2.
